# Integrated data analysis reveals significant associations of *KEAP1* mutations with DNA methylation alterations in lung adenocarcinomas

**DOI:** 10.18632/aging.103068

**Published:** 2020-04-23

**Authors:** Mohamed Elshaer, Ahmed Islam ElManawy, Ahmed Hammad, Akhileshwar Namani, Xiu Jun Wang, Xiuwen Tang

**Affiliations:** 1 Department of Biochemistry and Department of Thoracic Surgery of the First Affiliated Hospital, Zhejiang University School of Medicine, Hangzhou 310003, PR China; 2 College of Biosystems Engineering and Food Science, Zhejiang University, Hangzhou 310058, PR China; 3 Department of Pharmacology and Cancer Institute, The Second Affiliated Hospital, Zhejiang University School of Medicine, Hangzhou 310009, PR China; 4 Labeled Compounds Department, Hot Labs Center, Egyptian Atomic Energy Authority, Cairo 13759, Egypt; 5 Agricultural Engineering Department, Faculty of Agriculture, Suez Canal University, Ismailia 41522, Egypt; 6 Radiation Biology Department, National Center for Radiation Research and Technology, Egyptian Atomic Energy Authority, Cairo 13759, Egypt

**Keywords:** KEAP1, NRF2, DNA methylation, TCGA, lung cancer

## Abstract

*KEAP1* regulates the cytoprotection induced by NRF2 and has been reported to be a candidate tumor suppressor. Recent evidence has shown that mutations in several driver genes cause aberrant DNA methylation patterns, a hallmark of cancer. However, the correlation between *KEAP1* mutations and DNA methylation in lung cancer has still not been investigated. In this study, we systematically carried out an integrated multi-omics analysis to explore the correlation between *KEAP1* mutations and DNA methylation and its effect on gene expression in lung adenocarcinoma (LUAD). We found that most of the DNA aberrations associated with *KEAP1* mutations in LAUD were hypomethylation. Surprisingly, we found several NRF2-regulated genes among the genes that showed differential DNA methylation. Moreover, we identified an 8-gene signature with altered DNA methylation pattern and elevated gene expression levels in LUAD patients with mutated *KEAP1*, and evaluated the prognostic value of this signature in various clinical datasets. These results establish that *KEAP1* mutations are associated with DNA methylation changes capable of shaping regulatory network functions. Combining both epigenomic and transcriptomic changes along with *KEAP1* mutations may provide a better understanding of the molecular mechanisms associated with the progression of lung cancer and may help to provide better therapeutic approaches.

## INTRODUCTION

Lung cancer is the leading cause of cancer deaths worldwide. Human lung cancers are classified into two major histologic types, small-cell lung cancer and non-small-cell lung cancer (NSCLC), the latter comprising several subtypes. Previously, lung squamous cell carcinoma was the predominant form of NSCLC, but in the last few decades it has been replaced by lung adenocarcinoma (LUAD). Moreover, LUAD is the most common type of lung cancer in women, non-smokers, and young people [1].

Epigenetic changes in tumor tissue are involved in the pathogenesis of cancer. DNA methylation is a well-studied epigenetic alteration in cancer, owing in part to recent developments in techniques for genome-wide DNA methylation profiling [2, 3]. DNA methylation is the covalent addition of a methyl group to the 5^th^ carbon of the cytosine base within the cytosine-guanine (CpG)-dinucleotide in DNA. This has been shown to change the chromatin structure and affect the binding of transcription factors to DNA, and may thus regulate gene expression [4]. Recently, a novel DNA modification, N-6 methylated deoxyadenosine (m6dA), has been discovered in eukaryotic genomes [5]. Despite its low abundance in eukaryotes, m6dA is implicated in human diseases such as cancer. In NSCLC, several DNA methylation changes have been reported in association with neoplastic transformation, and some have been proposed as potential biomarkers with clinical relevance for diagnosis, prognosis, and response to therapy [6].

The *KEAP1*–NRF2 pathway plays a critical role in oxidative stress responses by triggering antioxidant and anti-inflammatory effects [7, 8]. In healthy tissue, *KEAP1* counteracts NRF2 by leading to its degradation [9, 10]. After exposure to oxidative stress, *KEAP1* is inactivated and no longer able to bind and control NRF2, which is subsequently stabilized and translocated into the nucleus [11, 12]. There, NRF2 promotes the transcription of genes encoding detoxification enzymes and antioxidant proteins [13].

Although cytoprotection by NRF2 activation is important for cancer chemoprevention in normal and pre-malignant tissues, in fully malignant cells NRF2 activity provides a growth advantage by increasing chemoresistance and enhancing tumor cell growth [14]. Somatic mutations in *KEAP1* lead to activation of the NRF2 signaling pathway in various cancers. Constitutively abundant NRF2 protein causes the increased expression of genes involved in drug metabolism, thereby increasing the resistance to chemotherapeutic drugs and radiotherapy [15]. In addition, over-expression of NRF2 also promotes cell proliferation and metastasis [16].

In this study, we analyzed TCGA (The Cancer Genome Atlas) 450k methylation data of LUAD patients in order to identify alterations of DNA methylation pattern associated with *KEAP1* mutations. Furthermore, we used TCGA RNA-Seq gene expression data of LUAD patients to investigate the association of these DNA methylation changes with gene expression. Eventually, we identified 8-gene biomarker for LUAD patients with mutated *KEAP1* based on aberrant DNA methylation associated with *KEAP1* mutations, and we evaluated the prognostic value of the 8-gene biomarker in several clinical datasets.

## RESULTS

The main goal of this study is to identify DNA methylation and gene expression changes associated with *KEAP1* mutations in LUAD. In order to achieve this goal, LUAD patient samples that have gene mutation, DNA methylation and gene expression data were required. The only source that provides such data for the same patient simultaneously is TCGA. Therefore, we conducted our study using TCGA data and we divided the patients into two groups, discovery cohort and another group to validate (validation cohort) the findings from the discovery cohort. We checked the TCGA–LUAD mutation data using the UCSC Xena browser and found that, of 544 LUAD patients with mutation data, only 98 had *KEAP1* mutations (18%). We also found that only 77 of 98 *KEAP1*-mutant patients had both DNA methylation and gene expression data. TCGA published the mutation and gene expression data of 230 LUAD samples in 2014 [17]. Of these 230 LUAD samples, we found 185 samples have DNA methylation data (30 with *KEAP1* mutations and 155 have no *KEAP1* mutation) and we considered this group of patients as our discovery cohort. Then, we randomly selected another 185 LUAD patients (30 have *KEAP1* mutations and 155 have no *KEAP1* mutation) with both DNA methylation and gene expression data and we considered this group of patients as our validation cohort. In addition, the frequency of mutation of other common driver genes of LUAD, such as TP53 and KRAS, was maintained very close between the *KEAP1*-mutated and wild-type groups. Therefore, none of these driver genes was considered as a variable that can contribute to methylation changes between the two groups. Also, all the patients included in the two groups have smoking history. So, this eliminated smoking as a contributing factor for any methylation changes found between the two groups.

### Overview of *KEAP1* mutations in LUAD

**Figure 1:**
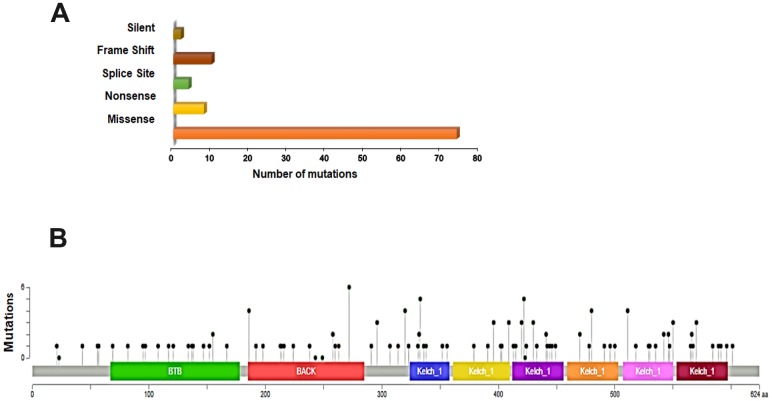
**Overview of genetic changes in *KEAP1* in TCGA–LUAD patients.** (**A**) Bar chart showing the types and numbers of mutations of *KEAP1*. (**B**) OnkoKB-predicted mutation maps (lollipop plots) showing the locations of mutations in the functional domains of *KEAP1* proteins. The lollipops show the locations of the mutations as identified by whole-exon sequencing.

In order to better understand the mutational landscape of *KEAP1* in LUAD, we used the USCS Xena browser to examine the types of mutations and their positions in the domain structure of *KEAP1* protein. We found that 18% (98 of 544) of the patient samples had *KEAP1* mutations. Among these, 75.5% (74 of 98) had missense mutations, while 8.1% (8 of 98) were nonsense mutations, 4.1% (4 of 98) were splice site mutations, 10.2% (10 of 98) were frame-shift mutations, and 2% (2 of 98) were silent mutations (Figure 1A). *KEAP1* consists of 605 amino-acids, and 3 main domains with 22 mutations have been reported in the BTB (broad-complex, tramtrack, and bric-a-brac) domain, 21 in the IVR (intervening region), and 42 in the Kelch domain (Figure 1B). Our discovery dataset had 30 patient samples with *KEAP1* mutations: 26 had missense mutations while 4 had truncating mutations.

### Aberrant DNA methylation is associated with *KEAP1* mutation in LUAD patients

**Figure 2:**
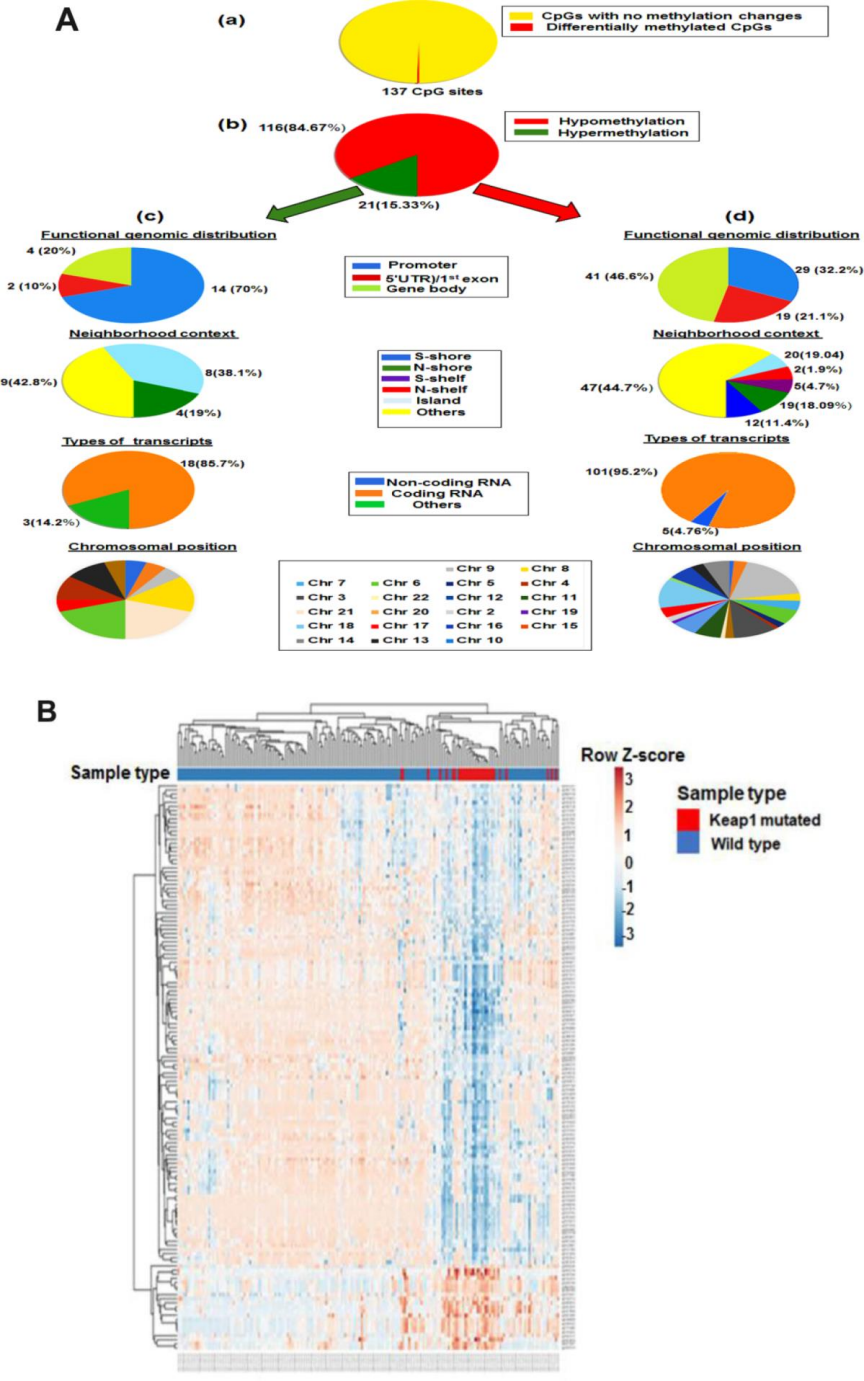
**Differential methylation analysis of *KEAP1*-mutated *vs* wild-type LUAD patients.** (**A**) Graphic showing 450k DNA methylation analysis. (**a**) Differentially-methylated CpG sites in *KEAP1*-mutated *vs* wild-type patient samples. (**b**) Percentages of hypomethylation and hypermethylation. (**c**) Distribution of hypermethylated CpG sites in *KEAP1*-mutated patient samples according to functional genomic distribution, neighborhood context, associated RNA transcripts, and chromosomal location. (**d**) Distribution of hypomethylated CpG sites in *KEAP1*-mutated patient samples according to functional genomic distribution, neighborhood context, associated RNA transcripts, and chromosomal location. (**B**) Heatmap showing the differentially-methylated CpG sites in *KEAP1*-mutated LUAD patients compared to their wild type counterparts (delta β >|0.2|, *p*<0.05 with BH-FDR adjustment). Methylation Beta-values are represented as row Z-score. The blue color indicates decreased methylation of CpG while the pink color indicates increased methylation of CpG. The heatmap was generated using the ClustVis webtool.

In order to identify the association of *KEAP1* mutations with DNA methylation in LUAD patients, we explored the differentially-methylated CpG sites between the *KEAP1*-mutated (30 patient samples) and wild-type (155 patient samples without *KEAP1* mutations) LUAD samples. A total of 137 differentially-methylated CpG sites were found between *KEAP1-*mutated and wild-type tumor samples after Benjamini Hochberg-false discovery rate (BH-FDR) adjustment (delta β > |0.2|, *P* <0.05) (Figure 2A, a). We found that 84.67% (116 of 137) of the CpG sites were hypomethylated, while 15.33% (21 of 137) were hypermethylated (Supplementary File 3) (Figure 2A, b). These findings suggest that most of the DNA methylation changes associated with *KEAP1* mutations are hypomethylation. To gain insight on this pattern, we divided the probes according to genomic localization, types of transcripts and chromosomal position. Genomic regions were divided into CpG islands which are genomic regions with high frequency of CpG sites; S-shores (regions up to 2 kb from CpG island in the south direction); N-shores (regions up to 2 kb from CpG island in the north direction); S-shelves (regions from 2 to 4 kb from CpG island in the south direction); N-shelves (regions from 2 to 4 kb from CpG island in the north direction) and open sea, in other words, the rest of the genome. Of these 116 hypomethylated CpG sites we were able to annotate 105 to the reference genome and found that 47 (44.7%) were in open sea, 20 (19.04%) in island, 19 (18.09 %) in S-shore,12 (11.4%) in N-shore, 5 (4.7%) in S-shelf, and 2 (1.9%) in N-shelf; in addition, 100 (95.2%) were in coding RNA-transcribed regions, while only five (4.76%) were in non-coding RNA transcribed-regions. The hypomethylated CpGs were functionally distributed as follow: 29 CpG (32.2%) were in promoter region, 19 (21.1%) were in 5′ untranslated region (UTR)/1^st^ exon, while 41 (46.6%) were in gene body. Among the 21 hypermethylated CpG sites, 14 CpG (70%) were in promoter region, 2 (10%) were in 5′ UTR)/1^st^ exon, while 4 (20%) were in gene body. In addition, 9 (42.8%) were in open sea, 8 (38.1%) in island, and 4 (19%) in N-shore. Besides, 18 of these hypermethylated CpG sites were in coding RNA-transcribed regions (Figure 2A, c).

The chromosomal distribution of both hypo and hyper methylated CpG sites were listed in Supplementary Table 1. Our data showed that DNA methylation in the *KEAP1*-mutated lung adenocarcinoma compared to their wild-type counterparts followed a distinct pattern along the gene coding sequence, with lower methylation levels near the transcription start site and gene body while higher methylation levels were mostly detected near the transcription start site. In addition, majority of hypomethylated CpGs were in open sea while majority of hypermethylated CpGs were in open sea and island. Moreover, the differentially-methylated CpG sites were embedded into 96 genes. Gene ontology (GO) and Kyoto Encyclopedia of Genes and Genomes (KEGG) pathway analyses showed that the differentially-methylated genes were enriched in biological processes and pathways related to cellular response to oxidative stress such as glutathione metabolism. The methylation profiles of 30 *KEAP1*-mutated and 155 wild-type LUAD patient samples were visualized on a heatmap produced by unsupervised hierarchical clustering. Major differences between the DNA methylation patterns enabled cluster analysis to discriminate between sample types. Significant differences or trends between *KEAP1*-mutated and non-*KEAP1*-mutated LUAD patient samples were detectable at 137 loci (Figure 2B). In addition, the overall methylation difference between *KEAP1*-mutated and non-*KEAP1*-mutated LUAD patient samples was highly significant.

**Figure 3:**
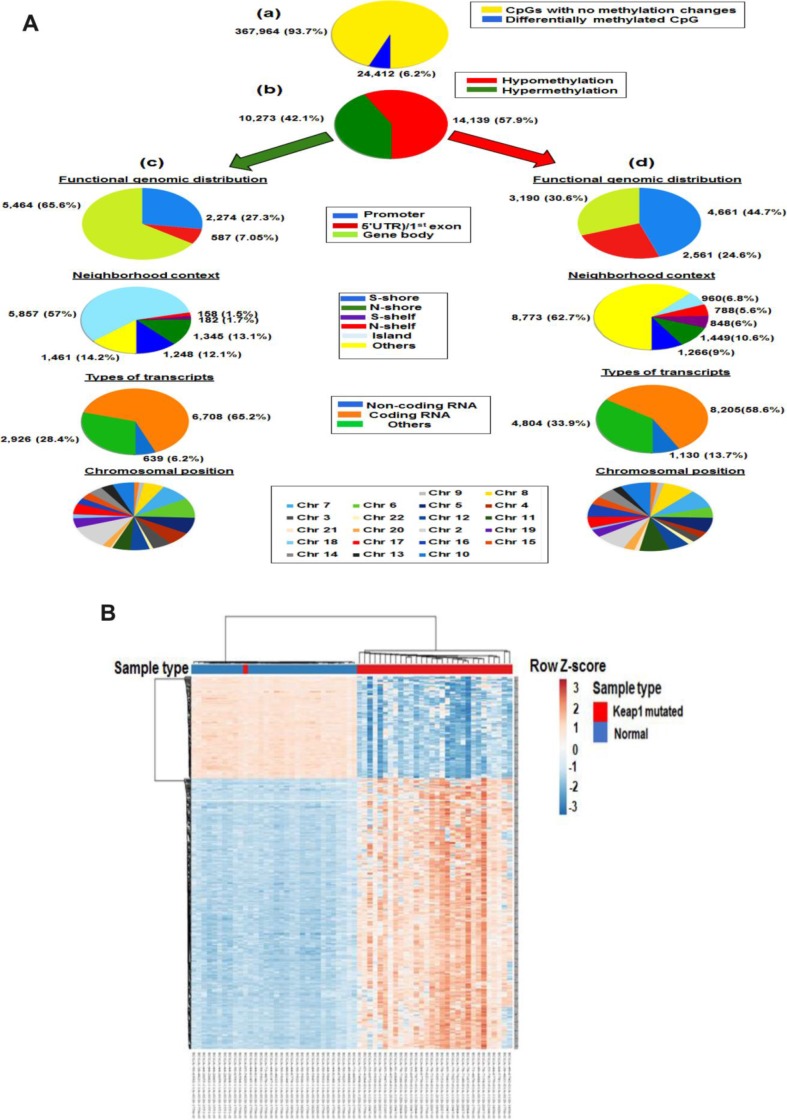
**Differential methylation analysis of samples from *KEAP1*-mutated LUAD patients *vs* normal samples.** (**A**) Graphic showing 450k DNA methylation analysis. (**a**) Graphic showing percentage of differentially-methylated CpG sites in *KEAP1*-mutated LUAD patients compared to normal samples. (**b**) Percentages of hypomethylation and hypermethylation. (**c**) Distribution of hypermethylated CpG sites in *KEAP1*-mutated patient samples according to functional genomic distribution, neighborhood context, associated RNA transcripts, and chromosomal location. (**d**) Distribution of hypomethylated CpG sites in *KEAP1*-mutated patient samples according to functional genomic distribution, neighborhood context, associated RNA transcripts, and chromosomal location. (**B**) Heatmap showing the differentially-methylated CpG sites in *KEAP1*-mutated LUAD patients compared to normal individuals (delta β >|0.4|, *p* <0.05 with BH-FDR adjustment). Methylation Beta-values are represented as row Z-score. The blue color indicates decreased CpG methylation while the pink color indicates increased CpG methylation. The heatmap was generated using the ClustVis webtool.

To validate the association of *KEAP1* mutation with the methylation of these CpG sites, we subjected the *KEAP1*-mutated versus normal samples to differential methylation analysis. In this data set, a total of 24,412 CpG sites were found to be differentially methylated after FDR adjustment (delta β >|0.2|, *P* <0.05) (Figure 3A, a). Of these sites, 10,273 were hypermethylated and 14,139 were hypomethylated (Figure 3A, b) (Supplementary File 4). Among the hypermethylated CpG sites, we found that 1,461 (14.2%) were in open sea, 5,857 (57%) in island, 1,248 (12.1%) in S-shore, 1,345 (13.1%) in N-shore, 182 (1.7%) in S-shelf, and 158 (1.5%) in N-shelf; in addition, 6,708 (65.2%) were in coding RNA-transcribed regions, while 639 (6.2%) were in non-coding RNA-transcribed regions (Figure 3A, c). The hypermethylated CpGs were functionally distributed as follow: 2,274 CpG (27.3%) were in promoter region, 587 (7.05%) were in 5′UTR)/1^st^ exon, while 5,464 (65.6%) were in gene body. Among the hypomethylated sites, 4,661 CpG (44.7%) were in promoter region, 2,561 (24.6%) were in 5′UTR)/1^st^ exon, while 3,190 (30.6%) were in gene body. In addition, 8,773 (62.7%) were in open sea, 960 (6.8%) in island, 1,266 (9%) in S-shore, 1,449 (10.6%) in N-shore, 848 (6%) in S-shelf, and 788 (5.6%) in N-shelf; in addition, 8,205 (58.6%) were in coding RNA-transcribed regions, while 1,130 (13.7%) were in non-coding RNA-transcribed regions (Figure 3A, d). The chromosomal distribution of both hypo and hyper methylated CpG sites were listed in Supplementary Table 2. Overall, DNA methylation in *KEAP1*-mutated lung adenocarcinoma compared to normal tissues followed a distinct pattern along the gene coding sequence, with lower methylation levels mostly near the transcription start site and higher methylation levels mostly at gene body. In agreement with various cancers, majority of hypermethylated CpGs were in island while majority of hypomethylated CpGs were in open sea [18, 19].

Altogether, the data obtained from the *KEAP1*-mutated lung adenocarcinoma followed the previous reports of Genome-wide DNA methylation patterns in lung cancer [19, 20]. In addition, *KEAP1*-mutated lung adenocarcinoma showed distinctive DNA methylation pattern which make it distinguishable from their wild-type counterparts.

Also, we used unsupervised hierarchical clustering to visualize the major differences between the DNA methylation patterns of 30 *KEAP1*-mutated LUAD patient samples and 32 from normal individuals. The heatmap showed clustering of the top 521 differentially-methylated loci (Figure 3B).

**Figure 4:**
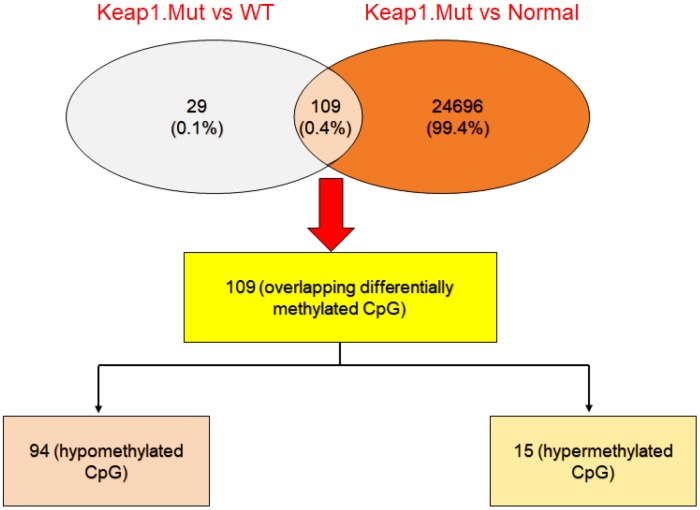
**Integrative analysis to cross-check the differentially-methylated CpG sites in *KEAP1*-mutated LUAD.** Venny diagram showing the differentially-methylated CpG sites overlapping between *KEAP1*-mutated *versus* wild-type and *KEAP1*-mutated *versus* normal datasets.

Then, integration analysis was performed between the differentially-methylated CpG sites found in the mutated *KEAP1* versus the wild-type and mutated *KEAP1* versus normal datasets in order to identify overlapping differentially-methylated CpG sites. We found 109 common differentially-methylated CpG sites (representing 80 genes) (Figure 4). Interestingly, 94 of these sites were hypomethylated and 15 were hypermethylated. Surprisingly, we found several differentially-methylated CpG sites that belonged to several *bonafide* NRF2 target genes such as *GPX2, TXNRD1, GCLC, PGD, SRXN1, AKR1C1, AKR1C2, ABCC1, ABCC2, MAFG, and SQSTM1* (Table 1). *KEAP1* dysfunction and increased NRF2 accumulation in the nucleus have been frequently reported in lung cancer. Moreover, changes in the *KEAP1*–NRF2 pathway and their association with tumor progression, resistance to chemotherapeutic drugs, and poor prognosis have been well documented [21]. In addition, we found cg10880599, cg04909257, cg04806177, cg23230478, cg00926657, cg03331715, cg02731193, and cg19648686 that belonged to the *GPX2, PGD, MSRB1, ACOT7, MAFG, TXNRD1, GCLC,* and *AKR1C1* genes, respectively, in the top list of hypomethylated CpG sites. On the other hand, we found Cg25693302 and Cg15733882 of the *NEDD4L* gene as well as Cg02370667 and Cg22167353 of the *NECAB2* gene, besides Cg06632214 and Cg00474080 that belonged to the *IFITM1* and *HCG21* genes, respectively, in the top list of hypermethylated CpG sites.

**Table 1:** The top listed differentially-methylated CpG sites between *KEAP1*-mutated and wild-type LUAD patient samples.

**Hypomethylated CpG sites**				**Hypermethylated CpG sites**			
**CpG name**	**Gene symbol**	**Delta Beta**	**P value**	**CpG name**	**Gene symbol**	**Delta Beta**	**P value**
cg10880599	GPX2	-0.38	1.60E-10	Cg25693302	NEDD4L	0.275	3.2488E-11
cg04909257	PGD	-0.36	3.11E-09	Cg06632214	IFITM1	0.259	2.0889E-05
cg04806177	MSRB1	-0.35	1.94E-07	Cg02370667	NECAB2	0.241	2.7964E-07
cg23230478	ACOT7	-0.348	7.81E-08	Cg15733882	NEDD4L	0.231	2.1958E-10
cg00926657	MAFG	-0.343	2.79E-09	Cg22167353	NECAB2	0.229	3.7118E-07
cg03331715	TXNRD1	-0.3437	5.91E-09	Cg00474080	HCG21	0.222	3.5719E-05
cg02731193	GCLC	-0.340	7.64E-08	Cg15245625	NEDD4L	0.220	3.6348E-12
cg19648686	AKR1C1	-0.333	5.60E-09	Cg00225623	SPATA13	0.214	2.6977E-06
cg19648686	AKR1C2	-0.333	5.60E-09	Cg04579966	SPATA13	0.213	4.7484E-05
cg09643186	GPX2	-0.330	1.78E-09	Cg14186816	NEDD4L	0.212	2.8999E-11
cg26155983	GPX2	-0.321	5.74E-08	Cg13656752	STK10	0.210	1.6346E-06
cg09489844	MAFG	-0.320	2.31E-09	Cg03030717	TBC1D30	0.203	0.00037491
cg18484212	SRXN1	-0.310	1.30E-06	Cg08781140	MYO15B	0.202	0.00014414
cg23012192	SQSTM1	-0.305	1.07E-06	cg19378330	ABCC2	0.201	9.8216E-05
cg20372666	HK1	-0.285	8.62E-10				
cg04020792	ATP2A2	-0.277	2.02E-05				
cg19509829	ATP2A2	-0.268	4.97E-06				
cg14420550	RP11-146I2.1	-0.262	2.11E-07				
cg18496841	DLGAP2	-0.254	6.94E-07				
cg01189072	CDK11B	-0.250	4.45E-05				
cg05116443	DNAJC5	-0.249	2.58E-06				

*In silico* analysis identifies the known and putative NRF2 binding sites in the promoter regions of differentially-methylated genes

**Figure 5:**
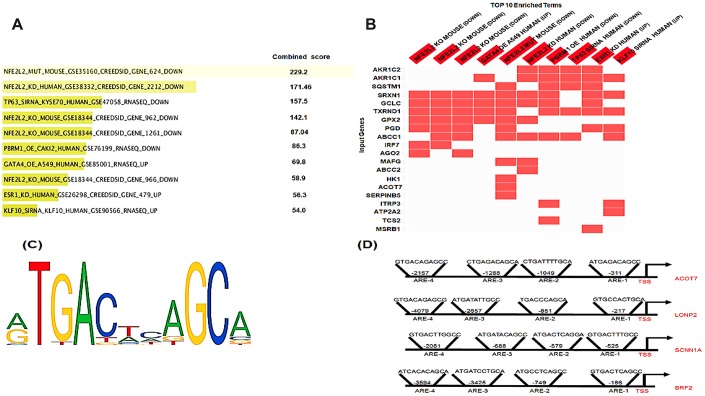
***In silico* analysis of NRF2 binding sites.** (**A**) Bar graph showing the top 10 enriched transcription factor perturbation followed by expression datasets (highest p value) after the input of the 80 differentially methylated genes using enrichr database. 5 data sets where NRF2 was Perturbed among the top 10 enriched terms (highest p value). (**B**) Heatmap showing the differentially-methylated genes (20 genes) that were enriched in any of the top 10 enriched terms. The heatmap shows 15 out of these 20 genes enriched in 5 datasets where NRF2 was Perturbed. (**C**) The NRF2 binding motif as provided by JASPER. (**D**) Schematic representation of the locations of *in silico*-predicted NRF2 binding sites (AREs) in the promoter regions of the human *ACOT7*, *LNOP2*, *SCNN1A*, and *BRF2* genes.

As *KEAP1* mutations lead to enhanced NRF2 activity, we investigated whether the differentially-methylated genes are potential NRF2 targets. To this end, we used a transcription factor perturbation-related gene expression database – enrichr (http://amp.pharm.mssm.edu/Enrichr/) – to determine whether 80 of these differentially-methylated genes were downregulated when NRF2 was knocked out, knocked down, or mutated in different cell lines and mouse models (Figure 5A, 5B). Intriguingly, 22 of the 80 differentially-methylated genes were downregulated. Further, we found that 11 of them were known NRF2 target genes. To identify the putative and known antioxidant responsive elements (AREs) (Figure 5C) in the other 11 genes, we used the LASAGNA-Search 2.0 web tool [22]. Interestingly, as Figure 5D showed positions of NRF2 binding sites (AREs) in the promoter regions of human *ACOT7, LNOP2, SCNN1A,* and *BRF2* genes, *in silico* analysis identified putative ARE sequences within the –5 kb upstream promoter regions of all 11 genes (Supplementary File 5).

### Differentially-expressed genes (DEGs) associated with *KEAP1* mutation in LUAD patients

**Figure 6:**
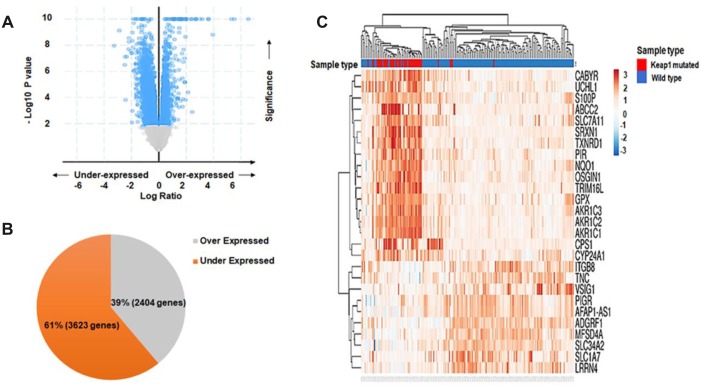
**Differential gene expression analysis.** (**A**) Volcano plot showing the distribution of DEGs between *KEAP1*-mutated and wild-type LUAD patient samples based on significance and fold change. (**B**) Pie chart showing the percentages of overexpressed and underexpressed genes. (**C**) Heatmap showing the top DEGs between *KEAP1*-mutated and wild-type LUAD patient samples with Log Fc>|2| and p <0.05.

In order to investigate the effect of changes in DNA methylation associated with *KEAP1* mutations on gene expression, we subjected the discovery data set (30 *KEAP1*-mutated *versus* 155 wild-type tumor samples) to DEG analysis. A total of 6,026 differentially-expressed genes (*P* <0.05) were identified (Supplementary File 6). Of these, we found that 2,404 genes were upregulated while 3,622 were downregulated (Figure 6A, 6B). Then, unsupervised hierarchical clustering was applied using the ‘ClustVis’ tool. The major differences between the gene expression patterns of *KEAP1*-mutated and wild-type LUAD patient samples enabled cluster analysis (Figure 6C). The heatmap showed clustering of the top DEGs with log Fc> |2|. Then, we integrated the differentially-methylated genes (β>|0.2|, *P* <0.05) with the DEGs (*P* <0.05), and found 36 overlapping genes with 50 differentially-methylated CpG sites. In addition, 30 (38 CpG sites) of these 36 genes were hypomethylated, while the remaining 6 (12 CpG sites) were hypermethylated (Supplementary File 7).

It has been reported that *KEAP1* mutations lead to the overexpression of NRF2 and its downstream genes in lung cancer. Thus, it was not surprising that we found many NRF2 target genes among the top list of upregulated genes in *KEAP1*-mutated LUAD patients with log Fc >1.5, including *NQO1*, *GCLM*, *GCLC*, *AKR1C3*, *AKR1C2*, *AKR1C1*, *GPX2*, *TXNRD1*, *ABCC2*, *G6PD*, *UGDH*, *TRIM16*, *TRIM16L*, *PGD*, *CY924A1*, and *OSGIN1*. Surprisingly, we found that several of the NRF2-regulated genes in this list were differentially- methylated (*GCLC*, *GPX2*, *TXNRD1*, *AKR1C*, *AKR1C2*, *SRXN1*, *PGD*, and *ABCC2*). Most of these had hypomethylated CpG sites, except for *ABCC2* that was hypermethylated at a CpG site within the gene body. These findings suggest that DNA methylation alterations associated with *KEAP1* mutations may synergize with NRF2 in regulating gene expression. However, we found 9 known NRF2 target genes (*NQO1*, *GCLM*, *AKR1C3*, *G6PD*, *UGDH*, *TRIM16*, *TRIM16L*, *OSGIN1*, and *CY924A1*) that did not show any DNA methylation changes in *KEAP1*-mutated tumor samples. This suggested that these genes are primarily regulated by NRF2. Interestingly, *OPN3* showed hypomethylation and overexpression with a fold-change of 1.48. *OPN3* has not been reported as an NRF2 target gene and *in silico* analysis showed that it was not a putative NRF2 target gene. The molecular mechanism of *OPN3* overexpression remains unclear.

### Identification of gene signatures associated with *KEAP1* mutation in LUAD

**Figure 7:**
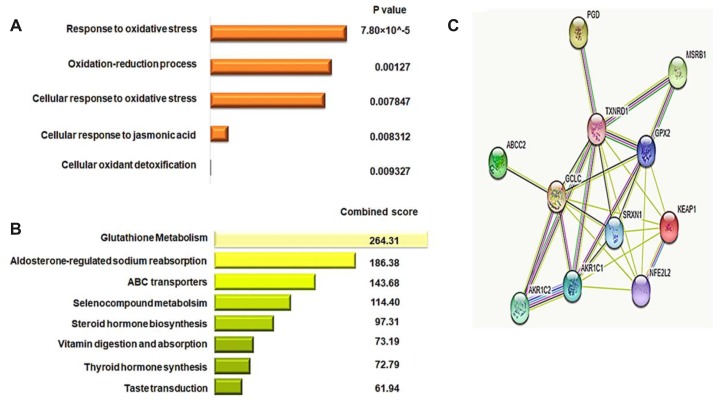
**Identification of the gene signature for *KEAP1*-mutated LUAD.** (**A**) Bar chart showing the top significant biological processes identified by GO analysis of 36 differentially-methylated genes using the DAVID webtool. (**B**) Bar chart showing the pathways with the top scores identified by KEGG pathway analysis of 36 differentially-methylated genes using the enrichr webtool. (**C**) Protein-protein interaction network analysis of the potential 9-gene signature predicting the functional correlation of the *KEAP1*–NRF2 axis with genes involved in drug metabolism and glutathione metabolic pathways in LUAD.

In order to identify a DNA methylation and gene expression signature for *KEAP1*-mutated LUAD, we first subjected the 36 genes that had differentially-methylated CpG sites to functional annotation analysis using DAVID (Database for Annotation, Visualization and Integrated Discovery) and enrichr. Functional annotation analysis from GO (Gene Ontology) and KEGG (Kyoto Encyclopedia of Genes and Genomes) pathway prediction using DAVID and enrichr, respectively, revealed that the 36 genes were enriched (*P* <0.01) in the following biological processes: Response to oxidative stress, Oxidation-reduction process, Cellular response to oxidative stress, Cellular response to jasmonic acid, and Cellular oxidant detoxification (Figure 7A, 7B). In the KEGG pathway analysis, we found enrichment (*P* <0.05) in pathways such as Glutathione metabolism, Aldosterone-regulated sodium reabsorption, ABC transporters, Selenocompound metabolism, Steroid hormone biosynthesis metabolism, Vitamin digestion and absorption, Thyroid hormone synthesis, and Taste transduction. Next, we selected the genes from the top three significant GO biological processes (Oxidative stress, Oxidation-reduction process, and Cellular response to oxidative stress). The selected genes were *GPX2*, *GCLC*, *TXNRD1*, *AKR1C1*, *AKR1C2*, *PGD*, *SRXN1*, *MSRB1*, *BRF2*, *ABCC2*, and *ATP2A2*. Then, we excluded *BRF2* and *ATP2A2* as they had an expression fold-change < 1. Eventually, 9 oxidative stress-related genes with 11 differentially-methylated CpG sites were obtained. Three of these sites belonged to *GPX2* (cg10880599, cg09643186, and cg26155983 were located in the gene body, 5′ untranslated region (UTR)/1^st^ exon, and within 1,500 bp upstream the transcriptional start site, respectively, and all were open sea), while *GCLC*, *TXNRD1*, *AKR1C1*, *AKR1C2*, *PGD*, *SRXN1*, *ABCC2*, and *MSRB1*each had only one differentially-methylated CpG site: cg02731193, cg03331715, cg19648686, cg19648686, cg04909257, cg18484212, cg19378330, and cg04806177, respectively. Most of these were located in the gene body, except for cg03331715 of *TXNRD1* that was in the 5′UTR. Moreover, *GCLC* and *MSRB1* had N-shore differentially-methylated CpG sites, while those belonging to *TXNRD1*, *ABCC2*, *AKR1C1*, and *AKR1C2* were open sea sites. Furthermore, *SRXN1* had an N-shelf differentially-methylated CpG, while that belonging to *PGD* was S-Shelf.

In addition to the GO and KEGG analyses, we used the STRING v10 database to construct a protein-protein interaction (PPI) network of the 9-gene potential signature along with the *KEAP1* and *NRF2* genes to reveal the complex associations between these genes. The enrichment results, based on the functional association between these genes, revealed that the majority were closely associated with each other through a coordinated interactive network (Figure 7C). Thus, PPI network analysis suggested that cross-talk of *KEAP1* and NRF2 with the 9-gene potential signature coordinately drives tumor progression and therapeutic resistance in LUAD.

**Figure 8:**
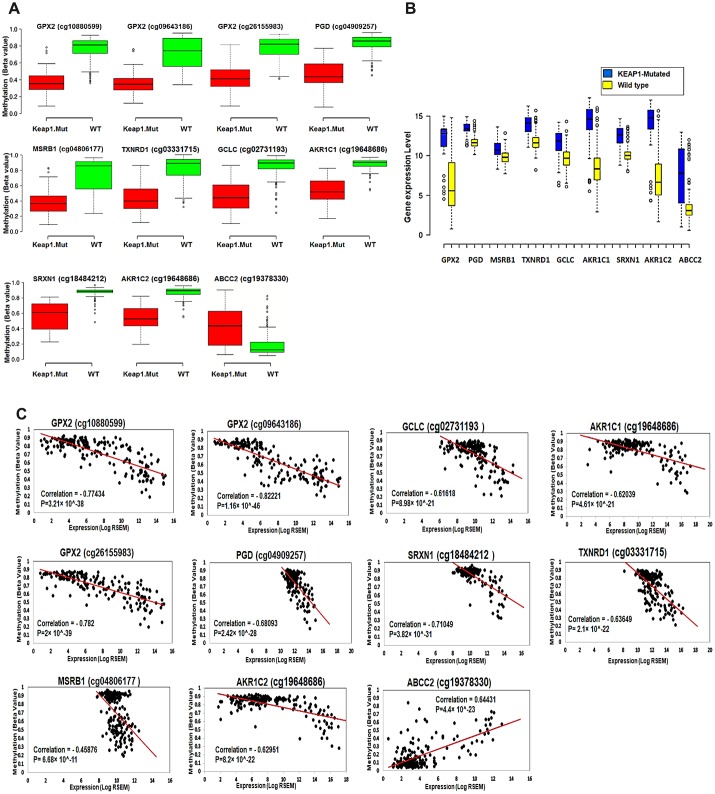
**Effect of DNA methylation changes at the 11 CpG sites on the expression of their corresponding genes.** (**A**) Box plots showing the differential methylation of 11 CpG sites between *KEAP1*-mutated and wild-type tumor samples. These 11 sites belong to the 9 genes included in the top 3 oxidative stress-related GO biological processes obtained by functional annotation analysis of the differentially-methylated genes (WT, wild-type; *KEAP1*.Mut, *KEAP1*-mutated). (**B**) Box plots showing the differential expression of the 9 differentially methylated genes between *KEAP1*-mutated and wild-type LUAD patient samples. Center lines show the medians; box limits indicate the 25^th^ and 75^th^ percentiles as determined by R software; whiskers extend 1.5 times the interquartile range from the 25^th^ and 75^th^ percentiles; outliers are represented by dots (n =100, 76, 16, 76, and 41 sample points). Boxplots were generated using BoxBlot R webtool. (**C**) Scatterplots showing the Spearman’s correlation between the methylation of the 11 CpG sites and the expression of their corresponding genes. A strong negative correlation can be seen between the methylation of all the CpG sites and the expression of their corresponding genes except for cg19378330 of the *ABCC2* gene, which shows a strong positive correlation.

The 11 CpG sites and their corresponding 9 genes showed significant differential methylation and gene expression between *KEAP1*-mutated and wild-type tumor samples (Figure 8A, 8B). In order to investigate the effect of methylation changes of these 11 CpG sites on the expression of their corresponding genes and examine the validity of these CpG sites as a basis for a gene expression signature specific for *KEAP1*-mutated LUAD, we applied Spearman’s correlation to the methylation beta values of these 11CpG sites and expression values of their corresponding genes across 185 LUAD patient samples. Interestingly, we found a strong negative correlation between methylation of the CpG sites cg10880599, cg09643186, and cg26155983 and the expression of *GPX2* as well as cg02731193, cg03331715, cg19648686, cg19648686, cg04909257, cg18484212, and cg04806177 and the expression of *GCLC*, *TXNRD1*, *AKR1C1*, *AKR1C2*, *PGD*, *SRXN1*, and *MSRB1*, respectively. However, we found a strong positive correlation between the methylation of cg19378330 and the expression of *ABCC2* (Figure 8C).

### Validation of DNA methylation and gene expression changes associated with *KEAP1* mutation in LUAD

To validate the DNA methylation alterations associated with *KEAP1* mutations in LUAD, we subjected the validation data set to differential methylation analysis (β>|0.2|, *p* <0.05) with BH-FDR adjustment. Interestingly, we found 626 differentially-methylated CpG sites with 582 hypomethylated and 44 hypermethylated (Supplementary File 8). This finding confirmed our result from the discovery data set that *KEAP1* mutation in LUAD was associated with DNA hypomethylation. Then, we specifically looked into the DNA methylation changes of these 11 candidates CpG sites. Consistent with our finding in the discovery data set, cg10880599, cg09643186, and cg26155983 that belonged to *GPX2*, as well as cg02731193, cg03331715, cg19648686, cg19648686, cg04909257, cg18484212, and cg04806177 that belonged to *GCLC*, *TXNRD1*, *AKR1C1*, *AKR1C2*, *PGD*, *SRXN1*, and *MSRB1*, respectively, showed hypomethylation (Table 2). Also, only cg19378330 of the *ABCC2* gene showed hypermethylation. Next, we used the LinkedOmics database to validate the overexpression of these 9 candidate genes in *KEAP1*-mutated LUAD. We applied differential gene expression analysis to 83 *KEAP1*-mutated and 478 wild-type LUAD patient samples using the LinkedOmics database (Supplementary File 9). Of these 9 candidate genes, 8 were significantly overexpressed with log Fc>1.5 (*GPX2*, *GCLC*, *TXNRD1*, *AKR1C1*, *AKR1C2*, *PGD*, *SRXN1*, and *ABCC2*). *MSRB1* was excluded from the *KEAP1* mutation-based gene signature as we could not find expression values for *MSRB1* in the LinkedOmics database. Therefore, the 8-gene represents a *KEAP1* mutation-based DNA methylation and gene expression signature for LUAD.

**Table 2:** The differential methylation of 11 CpG sites and the differential gene expression of their corresponding 9 genes in both discovery and validation data sets.

**CpG Name**	**Gene symbol**	**Delta beta values in discovery group**	**Delta beta value in validation group**	**Log F_C_ in discovery group**	**Log F_C_ in validation group**
cg10880599	GPX2	-0.379498	-0.34886	5.51	5.189
cg09643186	GPX2	-0.330261	-0.28731	5.51	5.189
cg26155983	GPX2	-0.321305	-0.2569	5.51	5.189
cg04909257	PGD	-0.359443	-0.22641	1.68	1.72
cg04806177	MSRB1	-0.348812	-0.24681	1	-
cg03331715	TXNRD1	-0.343764	-0.32573	2.9	2.15
cg02731193	GCLC	-0.340017	-0.23975	1.81	2.02
cg19648686	AKR1C2	-0.333994	-0.20816	6.45	6.22
cg19648686	AKR1C1	-0.333994	-0.20816	5.31	5.27
cg18484212	SRXN1	-0.310776	-0.23541	2.37	2.35
cg19378330	ABCC2	0.201648	0.234805	3.94	3.46

### The 8-gene signature is significantly associated with poor survival in LUAD patients

**Figure 9:**
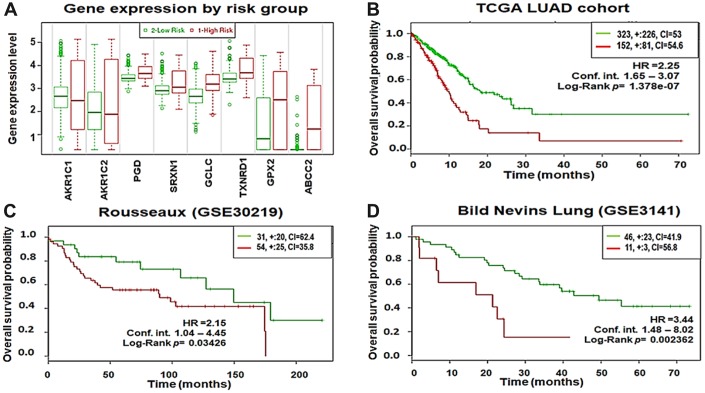
**Eight-gene signature predicts poor survival in three independent cohorts.** (**A**) Box plots showing the expression differences of the 8-gene signature in low (green) and high (red) risk groups of TCGA–LUAD patients (y-axis, gene expression value of each gene). (**B**) Kaplan-Meier survival plots showing that high expression of the 8-gene signature was associated with poor survival in TCGA–LUAD patients. (**C**) Rousseaux (GSE30219) cohort. (**D**) Bild Nevins Lung (GSE3141) cohort. Red, high-risk group; green, low-risk group; top right corner inset, numbers of high- and low-risk samples (+, numbers of censored samples) and concordance index (CI) of each risk group (x-axis, time (months); y-axis, overall survival probability; HR, hazard ratio; Conf.Int. confidence interval).

To evaluate the prognostic power of the 8-gene signature in patient survival, we first analyzed overall survival in the TCGA–LUAD cohort using the SurvExpress database. A total of 475 patient samples were divided into high-risk (n = 152) and low-risk groups (n = 323) based on their expression patterns (Figure 9A). The separation of risk groups was optimized using the ‘maximize risk group’ option provided in the SurvExpress database. The survival probability estimates in the two risk groups were visualized as Kaplan-Meier plots. Strikingly, overall survival analysis revealed that the patients in the high-risk group had poorer survival (HR = 2.25; CI = 60.38; p = 1.378×10^-7^) than the low-risk group (Figure 9B). Moreover, optimizing risk group separation of the Rousseaux (GSE30219) cohort (85 LUAD patient samples out of 264 lung cancer patient samples) showed that LUAD patients in the high-risk group (n=54) with increased expression of the 8-gene signature had poorer survival (HR = 2.15; CI = 53.48; *p* = 0.03426) than the low-risk group (n=31) (Figure 9C). In addition, we analyzed the overall survival in the Bild Nevins Lung (GSE3141) cohort available in the SurvExpress database. After optimized risk group separation, a total of 57 LUAD patient samples out of 255 lung cancer patient samples included in the cohort were divided into high-risk (n = 11) and low-risk groups (n = 46) based on their expression patterns (Figure 9D). The survival probability estimates in the two risk groups were represented as Kaplan-Meier plots. Similarly, overall survival analysis showed that the patients in the high-risk group had poorer survival (HR = 3.44; CI = 55.23; p = 0.002362) than the low-risk group.

## DISCUSSION

To the best of our knowledge, this is the first study to demonstrate the DNA methylation changes associated with *KEAP1* mutations and their effect on gene expression in LUAD patients. Mutations in the *KEAP1*^_^NRF2 pathway are known to be involved in malignant transformation in various cancer types [23]. It has been shown that *KEAP1* gene mutations occur in ~20% of NSCLCs [23]. *KEAP1* mutations lead to constitutively active NRF2, enhanced transcriptional induction of antioxidants, xenobiotic metabolism enzymes, drug efflux pumps, and the subsequent protection of cancer cells against chemotherapeutic drugs [24]. Here, we showed that 18% of the TCGA–LUAD patient samples had *KEAP1* mutations. However, a recent study on tumor tissue from 1,391 NSCLC patients using next-generation sequencing, reported *KEAP1* mutations in 157 patients (11.3%) and identified up to 134 different mutations [25]. Our analysis demonstrated that *KEAP1* mutations affected the DNA methylation pattern in LUAD, most of which were hypomethylation (84.67%), and 44.7% of the hypomethylation events occurred at open sea CpG sites. Consistent with our results, Chen et al., reported that *KEAP1* mutations in LUAD play a role in genome-wide open sea hypomethylation [26]. Somatic mutations of *TP53*, one of the well-studied tumor suppressors, are associated with DNA methylation changes. It has been reported that polymorphism at codon 72 rs1042522 of *TP53*, a polymorphism known to affect the somatic mutation rate in human carcinomas, is associated with higher DNA methylation [27]. Moreover, the DNA methylation landscape can be affected by mutations in epigenetic modifying enzymes such as *SETD2*, the H3K36me3 writer [28]. Furthermore, mutations in driver genes may perturb the transcriptional circuitry. Such perturbation can aberrantly activate or inactivate DNA-binding proteins causing DNA methylation changes near their binding sites [29, 30].

The locations of the differentially-methylated CpG sites between *KEAP1*-mutated and wild-type LUAD tumor samples varied among promoter, 5′UTR, 1^st^ exon, and gene body. In agreement with the findings of Varley et. al., we found that the correlation between CpG methylation and gene expression depends on the genomic context [31]

This is evidenced by the recent finding that *BRAF* V600E and *KRAS* G13D mutations in colon adenocarcinoma upregulate the transcription factors *MAFG* and ZNF304, respectively, resulting in targeted promoter CGI hypermethylation near the *MAFG* and ZNF304 binding sites [32, 33]. Finally, several biological processes that alter DNA methylation at specific sites have been documented recently, and driver gene mutations that promote these DNA methylation-altering processes may change DNA methylation at affected sites. For example, cellular oxidative stress produces hypermethylation at the promoters of low-expression genes [34]; hypoxia reduces 5-methyl-cytosine hydroxylase activity, leading to hypermethylation at targeted sites [35]; hypoxia reduces TET activity, leading to hypermethylation at targeted sites [35]; and cell proliferation causes aberrant DNA methylation to accumulate in the promoters of polycomb group target CpG. In addition, O′Hagan et al. demonstrated that induced cellular oxidative stress recruits DNA methyltransferase 1 (DNMT1) to damaged chromatin. DNMT1 becomes part of a complex(es) containing DNMT3B and members of polycomb repressive complex 4 [34]. Promoters enriched in this complex have histone mark changes and DNA hypermethylation which lead eventually to transcriptional silencing. Paradoxically, this finding by O′Hagan et al. could give an explanation for the noted DNA hypomethylation associated with *KEAP1* mutation in LUAD. As we noted earlier, *KEAP1* mutations in LUAD result in higher activity of NRF2 and the consequent overexpression of its downstream genes that play a major role in reducing cellular oxidative stress, which ultimately may result in hypomethylation of the reported CpG sites. GO and KEGG pathway analysis of the differentially-methylated genes in *KEAP1*-mutated LUAD patient samples identified biological processes and pathways involved in reducing oxidative stress. Also, this may suggest the presence of a positive feedback loop between cellular oxidative status and methylation of these CpG sites as well as the expression of their corresponding genes.

Also, we identified a 8-gene expression signature based on DNA methylation alterations associated with *KEAP1* mutations in LUAD patients. The 8 genes (*GPX2*, *GCLC*, *TXNRD1*, *AKR1C1*, *AKR1C2*, *PGD*, *SRXN1*, and *ABCC2*) showed methylation changes across one or more CpG sites. Further, we tested this signature in 3 independent clinical cohorts, including the TCGA–LUAD cohort. Kaplan-Meier survival plots generated for all 3 cohorts showed that higher expression of this gene signature is significantly correlated with poor survival outcomes.

Recently, it has been suggested that *GPX2* might be an important predictor for the prognosis of esophageal squamous cell carcinoma and a potential target for the intervention and treatment of this disease [36]. In addition, it has been shown that a high GCLC level in tumor tissue is associated with a poor prognosis of hepatocellular carcinoma after curative resection [37]. Also, high mRNA expression of GCLC in cancer tissue has been suggested as a potential predictor of cisplatin resistance in lung adenocarcinoma patients [38]. Moreover, *TXNRD1* overexpression has been reported in many human malignancies and functions as a prognostic factor for many tumors, such as oral squamous cell carcinomas, lung cancer, breast cancer, astrocytomas, and hepatocellular carcinoma [39]. Furthermore, Tian et al. indicated that *AKR1C1* plays an important role in the development and progression of small-cell lung cancer and may represent an independent biomarker for assessment of the primary prognosis and therapy of small-cell lung cancer [40]. In addition, it has been suggested that *AKR1C1* was related to drug resistance and targeting *AKR1C1* might be an alternative therapy method for SCLC patients [41]. Moreover, it has been stated that lowering the expression of *AKR1C1* may inhibit proliferation and migration leading to reduction of drug resistance; in particular, Silencing *AKR1C1* could suppress tumor growth and invasion, and also decrease the resistance to regular chemotherapy and radiotherapy [40]. Additionally, *AKR1C2* may be considered as one of the biomarkers that indicates cisplatin resistance and may be act as one of the effective molecular targets for rescuing cisplatin sensitivity [42]. In agreement with our finding, Namani et al, have identified a gene expression signature that includes *TXNRD1*, *AKR1C1*, *AKR1C2*, and *SRXN1* along with other genes in head and neck squamous cell cancer and non small cell lung cancer [43, 44]. Interestingly, they found that higher expression of this gene signature is associated with poor survival and drug resistance. Moreover, it has been reported that *SRXN1* promotes cell invasion, migration in cervical cancer via activating the Wnt/β-catenin signaling pathway, and could be a promising tool for the development of better therapeutic strategies for cancer prevention and treatment [45]. Recent work suggested that 6-phosphogluconate dehydrogenase (*6PGD*), the key enzyme of oxidative pentose phosphate pathway (PPP), could act as a potential therapeutic target to enhance chemosensitivity in cervical cancer [46]. In addition, Becard et al. identified *PGD* as a potentially important pro-metastatic driver gene in pancreatic ductal adenocarcinoma [47]. Several studies showed that single nucleotide polymorphisms (SNPs) of the *ABCC2* gene are associated with altered distribution, metabolism and elimination of a plethora of drugs in several types of cancer [48–50]. Furthermore, Tumor characterized by overexpressing *ABCC2* protein has shown regression in size upon antisense *ABCC2* expression in combination with chemotherapy due to chemosensitization; for instance, Tumor size decreased when adenovirus expressing antisense *ABCC2* has been directly injected into HepG2 tumors in nude mice [51]. Here, we identified for the first time a gene expression signature based on the DNA methylation alterations associated with *KEAP1* mutations in LUAD.

## CONCLUSIONS

**Figure 10:**
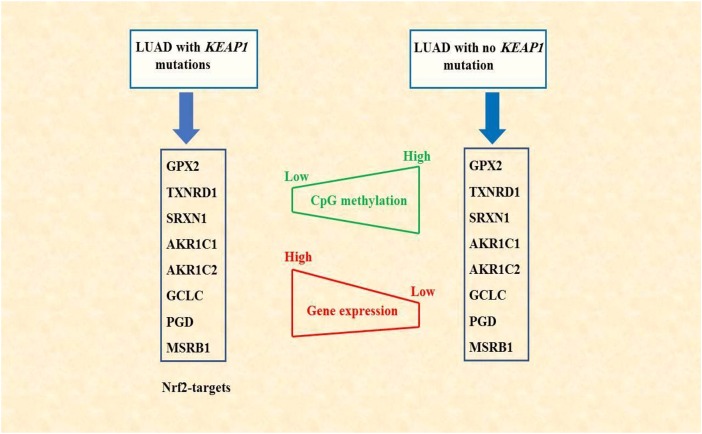
**Schematic diagram summarizing the findings of this study.** DNA methylation alterations associated with *KEAP1* mutations may synergize with NRF2 in regulating gene expression. NRF2-targets, the 8-gene signature, showed low CpG methylation but elevated gene expression levels in LAUD patients with mutated *KEAP1*.

In conclusion, we have described the changes in DNA methylation associated with *KEAP1* mutation in LUAD. In addition, we have illustrated a potential synergistic role between DNA methylation alterations associated with *KEAP1* mutations and the transcription factor NRF2 which eventually lead to the overexpression of some NRF2 target genes. This finding may provide a better understanding of the molecular mechanisms associated with lung cancer progression and drug resistance. Also, we have identified a signature based on DNA methylation aberration and gene expression for *KEAP1*-mutated LUAD (Figure 10). This signature may be used in the future as potential biomarker with clinical relevance for the diagnosis, prognosis, and response to therapy of LUAD.

## MATERIALS AND METHODS

### Overall database selection

The Cancer Genome Atlas (TCGA) human genome-wide DNA methylation Level 3 data (Illumina Infinium Human Methylation 450 K BeadChips Platform) for 460 LUAD along with 32 normal lung tissues as well as TCGA RNA-Seq gene expression version2 level 3 data (Illumina HiSeq platform) for 515 LUAD tissues were downloaded from the Broad GDAC (Global Data Assembly Centers) Firehose website (http://gdac.broadinstitute.org/). All the mutation data for *KEAP1* used in the present study was obtained from the UCSC Xena Browser (https://xenabrowser.net/) [52]. We checked the TCGA–LUAD mutation data using the UCSC Xena browser and found that, out of 544 LUAD patients with mutation data, only 98 had *KEAP1* mutations (18%). We also found that only 77 of these 98 *KEAP1*-mutant patients had both methylation and gene expression data (Supplementary File 1). Then, we segregated these patients into 3 datasets, the first had 185 LUAD patients [30 with *KEAP1* mutations (16.2%) and 155 without *KEAP1* mutations (wild-type)] and considered them to be the discovery dataset. The second dataset had 185 LUAD patients (30 with *KEAP1* mutations (16.2%) and 155 wild-type patients) and we marked this dataset as a validation cohort. The third dataset had 62 individuals (30 patients with *KEAP1* mutations from the discovery dataset and 32 normal individuals) (Supplementary File 2). The comparisons of either DNA methylation or gene expression were specifically held between *KEAP1*-mutated and wild-type (factors other than *KEAP1* mutations) tumor samples.

### DNA methylation analysis

We used TCGA–LUAD genome-wide methylation data (level 3) that were generated using the Illumina Infinium Human DNA Methylation 450 K array platform, which interrogates the methylation status of 480,000 CpG sites. DNA methylation levels were reported as β-values which represent the ratio of intensities between locus-specific methylated and unmethylated bead-bound probes. The β-value is a continuous variable, ranging from 0 (unmethylated) to 1 (fully methylated). The three data sets were analyzed in order to identify the differentially-methylated CpG sites between the different groups in each dataset. First, we filtered out probes containing single-nucleotide polymorphisms, repeat sequencing, and probes in the sex-chromosomes from all samples. Then we calculated the delta β-value for all the probes between the different groups. In order to identify differentially-methylated CpG sites, we applied Student’s t test with a *p*-value cutoff of < 0.05 and BH-FDR adjustment, and we also used a delta β-value cutoff of > |0.2|. Finally, in order to minimize false-positive results and crosscheck the DNA methylation changes associated with *KEAP1* mutation, we integrated the differentially-methylated CpG sites between both *KEAP1*-mutated *versus* wild-type and *KEAP1*-mutated *versus* normal datasets using Venny 2.1 (http://bioinfogp.cnb.csic.es/tools/venny/index.html).

### RNA-Seq data analysis

TCGA RNA-Seq gene expression version 2 level 3 data (Illumina Hiseq platform) for 515 LUAD tissues were used to subject the discovery dataset to differential gene expression analysis. Briefly, Level 3 transcriptomic data of the discovery dataset were normalized by the RSEM method [53]. All gene expression values were log-transformed to approximate the data to a normal distribution. The DEGs were identified by applying Student’s t-test with *P* < 0.05 (BH-FDR adjustment).

### Integrative analysis of DNA methylation and gene expression

In order to identify the effect of DNA methylation alterations associated with *KEAP1* mutations on gene expression, we performed an integrative analysis between genes that showed differential methylation owing to *KEAP1* mutation with a delta β-value > |0.2| and *P* value < 0.05 with BH-FDR adjustment, as well as genes that showed differential expression between *KEAP1*-mutated and wild-type patient samples with *P* < 0.05 (BH-FDR adjustment). The integrative analysis was performed using the Venny 2.1 web-based tool (http://bioinfogp.cnb.csic.es/tools/venny/index.html).

### Functional annotation and protein-protein interaction (PPI) network analysis

Functional annotation by GO and KEGG analyses of genes that shows differential methylation and differential expression simultaneously was performed using the updated version of the DAVID v6.8 web tool (https://david.ncifcrf.gov/) [54] and the enrichr database (http://amp.pharm.mssm.edu/Enrichr/) [55], respectively. In addition, PPI network analysis was performed using the STRING v10 database (https://string-db.org/) [56].

### Methylation and gene expression correlation analysis

All data were quantile normalized before correlation testing. Overall, 185 patient samples (30 *KEAP1*-mutated and 155 wild-type) were used to detect significant correlations. The correlation test statistic was based on Pearson’s correlation coefficient between DNA methylation beta values and gene expression levels.

### LinkedOmics database analysis

In order to validate the discovery dataset differential gene expression analysis, we carried out differential gene expression analysis between *KEAP1*-mutated and wild-type LUAD tumor samples using the LinkedOmics database (http://www.linkedomics.org/login.php) [57], an open access web-based resource that contains multi-omics data and clinical data for 32 types of cancer and a total of 11,158 patients from the TCGA project.

### Survival analysis

Cox proportional hazard regression was performed using the online survival analysis and biomarker validation tool SurvExpress(http://bioinformatica.mty.itesm.mx:8080/Biomatec/SurvivaX.jsp) [58]. We considered the data from a total of 617 LUAD patients in 3 independent cohorts available in the SurvExpress database: the TCGA–LUAD cohort (n = 475) with another two cohorts–Rousseaux et al., (GSE30219) (n = 86) [59], and Bild et al., (GSE3141) (n = 57) [60] for survival analysis. In the case of microarray-based survival data, we considered the average values for genes whose expression was associated with multiple probe sets such as duplicates or alternatives. SurvExpress separates the patient samples into two groups, high- and low-risk, based on the average expression of the 8-gene signature values, and performed statistical analysis of survival probability of the two groups using the log-rank method. SurvExpress implements two methods to generate risk groups. The first method (default) generates the risk groups splitting the ordered prognostic index (PI) (higher values for higher risk) by the number of risk groups leaving equal number of samples in each group. For two risk groups, this is equivalent to split the PI by the median. The second method to produce risk groups uses an optimization algorithm from the ordered PI. Briefly, for two groups, a log-rank test is performed along all values of the arranged PI. Then, the algorithm chooses the split point where the p-value is minimum. SurvExpress uses the log-rank test to generate Kaplan-Meir plots based on the ‘Survival’ package of the R platform, which is integrated into its website. Log-rank test *P* < 0.05 was considered to be statistically significant.

### Statistical analysis

Details of genome-wide analysis of methylation data are provided in the sections above.

*P* values for both differential CpG methylation and differential gene expression analyses were calculated using Student’s t-test. The *P* values were adjusted by the Benjamini-Hochberg procedure with an FDR< 0.05 (*q* values). Correlations between CpG methylation levels and the corresponding gene expression levels were calculated using Pearson’s correlation coefficient. All the statistical analyses were performed using the Python V3.7 programming language.

## Supplementary Material

Supplementary Tables

Supplementary File 1

Supplementary File 2

Supplementary File 3

Supplementary File 4

Supplementary File 5

Supplementary File 6

Supplementary File 7

Supplementary File 8

Supplementary File 9
