# Supplementary material for: Integrated data analysis reveals significant associations of KEAP1 mutations with DNA methylation alterations in lung adenocarcinomas
Source: Aging (Albany NY). 2020 Apr 23;12(8):7183–206. doi: 10.18632/aging.103068 (PMC7202502; doi:10.18632/aging.103068)
Supplement: Supplementary Tables [file aging-12-103068-s007..pdf]

SUPPLEMENTARY MATERIALS

Supplementary Files

Please browse Full Text version to see the data of Supplementary Files 1–9.

Supplementary File 1. TCGA *KEAP1*-mutated LUAD patient samples ID from Xena browser and ID of TCGA *KEAP1*-mutated patient samples that have both DNA methylation and gene expression data

Supplementary File 2. Discovery dataset (30 *KEAP1*-mutated vs 155 WT), Validation dataset (30 *KEAP1*- mutated vs 155 WT), and the third dataset (30 *KEAP1*- mutated vs 32 normal) patients ID.

Supplementary File 3. Differentially-methylated CpG sites from the discovery dataset (30 *KEAP1*-mutated vs 155 WT) with *p*-value cutoff of < 0.05 (BH-FDR adjustment), and a delta β-value cutoff of > |0.2|.

Supplementary File 4. Differentially-methylated CpG sites from the third dataset (*KEAP1*-mutated vs Normal with delta β-value > |0.2| and *p*-value < 0.05 (BH-FDR adjustment)).

Supplementary File 5. *insilico*-predicted NRF2 binding sites (AREs) in the promoter regions of 11 differentially-methylated genes using LASAGNA-Search 2.0 web tool.

Supplementary File 6. Differentially-expressed genes from the discovery dataset (30 *KEAP1*-mutated vs 155 WT) with *p*-value < 0.05 (BH-FDR adjustment).

Supplementary File 7. List of genes that shows both differential methylation and gene expression with *p*-value < 0.05 between *KEAP1*-mutated vs WT LUAD patient samples.

Supplementary File 8. Differentially-methylated CpG sites from the validation dataset (30 *KEAP1*-mutated vs 155 WT) with *p*-value cutoff of < 0.05 (BH-FDR adjustment), and a delta β-value cutoff of > |0.2|.

Supplementary File 9. List of differentially-expressed genes between *KEAP1*-mutated vs WT from Linkedomics database.

Supplementary Table 1. Chromosomal distribution of differentially-methylated CpG sites between *KEAP1*-mutated and wild-type LUAD samples.

Hypo methylated CpG sites		Hyper methylated CpG sites	
**Chromosome Number**	**Number of CpG sites**	**Chromosome Number**	**Number of CpG sites**
**1**	8	1	1
**2**	6	2	-
**3**	1	3	-
**4**	2	4	-
**5**	6	5	1
**6**	4	6	1
**7**	3	7	-
**8**	18	8	-
**9**	3	9	-
**10**	6	10	1
**11**	2	11	3
**12**	10	12	1
**13**	3	13	2
**14**	6	14	-
**15**	1	15	-
**16**	12	16	2
**17**	4	17	1
**18**	2	18	4
**19**	1	19	-
**20**	6	20	-
**21**	1	21	4
**22**	-	22	-

Supplementary Table 2. Chromosomal distribution of differentially methylated CpG sites between *KEAP1*-mutated LUAD and normal samples.

Hypo methylated CpG sites		Hyper methylated CpG sites	
**Chromosome Number**	**Number of CpG sites**	**Chromosome Number**	**Number of CpG sites**
**1**	1457	1	1063
**2**	1044	2	1010
**3**	341	3	473
**4**	364	4	594
**5**	884	5	720
**6**	641	6	885
**7**	1102	7	751
**8**	1050	8	503
**9**	208	9	127
**10**	994	10	532
**11**	1002	11	453
**12**	697	12	478
**13**	320	13	310
**14**	646	14	367
**15**	466	15	284
**16**	767	16	274
**17**	670	17	468
**18**	136	18	180
**19**	461	19	426
**20**	373	20	216
**21**	165	21	45
**22**	207	22	85
